# Effects of physical exercise on anxiety depression and emotion regulation in children with attention deficit hyperactivity disorder: a systematic review and meta-analysis

**DOI:** 10.3389/fped.2024.1479615

**Published:** 2025-01-07

**Authors:** Yagang Song, Shuqi Jia, Xing Wang, Aiwei Wang, Tao Ma, Shufan Li, Jiwei Chen, Zhaohui Guo, Feng Ding, Yuxi Ren, Man Qin

**Affiliations:** ^1^ Department of Physical Education Teaching, Shanghai Sanda University, Shanghai, China; ^2^ Physical Education, Shanghai University of Sport, Shanghai, China; ^3^ Physical Education, Yangzhou University, Yangzhou, China; ^4^ Faculty of Sports Science, Ningbo University, Ningbo, China; ^5^ School of Sports and Health, Shanghai Lixin Accounting and Finance University, Shanghai, China

**Keywords:** attention deficit hyperactivity disorder, physical exercise, anxiety, depression, emotional regulation, meta-analysis

## Abstract

**Objective:**

This systematic review and meta-analysis aimed to comprehensively evaluate the impact of physical exercise interventions on anxiety, depression, and emotional regulation in children diagnosed with attention deficit hyperactivity disorder (ADHD).

**Methods:**

A comprehensive search was conducted across multiple databases, including Embase, Web of Science (WOS), PubMed, The Cochrane Library, Wanfang Data, VIP Information, and China National Knowledge Infrastructure (CNKI), from their inception up to July 2024. The search aimed to identify randomized controlled trials (RCTs) investigating the impact of physical exercise on anxiety, depression, and emotional regulation in children diagnosed with ADHD. The Physiotherapy Evidence Database (PEDro) scale was employed to assess the quality of the literature, while the revised Cochrane risk-of-bias tool (ROB-2) was used to evaluate the overall risk of bias. The Grading of Recommendations, Assessment, Development and Evaluation (GRADE) profiler method was utilized to further assess the quality of evidence. Meta-analysis, sensitivity analysis, and publication bias testing were performed using Stata 18.0 software. Effect sizes were calculated using the standardized mean difference (SMD) and 95% confidence intervals (CI).

**Results:**

The analysis included 18 RCTs, encompassing 830 participants. Physical exercise exhibited a significant positive effect on anxiety (SMD = −0.58, *p* < 0.05), depression (SMD = −0.57, *p* < 0.05), and emotional regulation (SMD = 1.03, *p* < 0.05) in children diagnosed with ADHD. Subgroup analysis revealed that exercise programs with monotypic and mixed modalities, short duration, high frequencies, medium duration, and moderate intensities were the most efficacious in ameliorating anxiety symptoms. The mixed exercise program, when conducted for short duration, with low frequencies, medium duration, and moderate intensity was the most effective in alleviating depression symptoms. Exercise programs featuring mixed modalities, longer duration, moderate to high frequencies, shorter duration, and low intensity yielded the most significant improvements in emotional regulation.

**Conclusions:**

Research demonstrates that physical exercise mitigates anxiety and depression and improves emotional regulation in children with ADHD. A dose-response relationship is evident, correlating with the type, duration, intensity, frequency, and overall exercise duration.

**Systematic Review Registration:**

https://www.crd.york.ac.uk/prospero/, PROSPERO identifier (CRD42024571577).

## 1 Introduction

Attention-deficit/hyperactivity disorder (ADHD) is the most prevalent neurodevelopmental disorder in childhood (1), affecting approximately 7% of children globally, with an increasing prevalence annually (2, 3). ADHD is primarily characterized by inattention and hyperactivity/impulsivity disproportionate to age and developmental level, often accompanied by internalizing problems such as anxiety, depression, and emotional dysregulation (4). Approximately 25% to 50% of children with ADHD exhibit varying degrees of anxiety/depression and emotional dysregulation (5). Children with ADHD who experience emotional dysregulation are significantly more prone to anxiety/depression (6), increased attention deficit symptoms (7), and aggressive and disruptive behavior compared with other individuals with ADHD (8). Anxiety, depression, and emotional problems have become increasingly significant components of the core symptoms of ADHD (9), and affected children are at elevated risk for criminal activity (5), substance abuse (10), and other issues. If timely treatment and intervention are not provided, anxiety, depression, and emotional problems in children with ADHD may lead to higher levels of psychiatric comorbidity, greater social impairment, and increased persistence of ADHD symptoms (11). Consequently, a critical issue is determining effective intervention strategies to ameliorate anxiety, depression, and emotional problems in children with ADHD.

The conventional treatment for ADHD primarily involves medication (12). However, this approach is controversial due to various physiological side effects (13), including high blood pressure, sleep disturbances, and mood disorders (14–16). In recent years, non-pharmacological treatments such as cognitive behavioral therapy and parent training have gained popularity among ADHD clinicians (17, 18). Although these treatments avoid physiological side effects, they are time-consuming, making large-scale implementation challenging (19). Consequently, exercise is recommended as a safe, cost-effective, and easily implementable intervention for children with ADHD, serving as an adjunctive or complementary therapy (20). The effects of exercise intervention are comparable to those of medication (21). Meta-analyses have demonstrated that physical activity significantly improves attention deficit, hyperactivity/impulsivity, and executive functioning (22–24) in children with ADHD. However, research on the effects of physical activity on anxiety, depression, and emotional problems in children with ADHD remains limited and controversial.

The reviewed studies suggest that physical exercise can have varying effects on mental health outcomes, such as anxiety and depression, with results differing based on factors including intensity, duration, and the specific population examined. The meta-analysis results indicated that short-term aerobic exercise had a moderate to large effect on reducing anxiety (Cerrillo-Urbina, 2018) (14), while moderate-intensity physical training led to significant improvements in mental health problems (Sakshi, 2021) (24). However, another meta-analysis revealed no significant effect of exercise on depression (25). The randomized controlled trials (RCTs) generally demonstrated exercise to be beneficial in alleviating symptoms of anxiety and depression, although the effects varied with exercise intensity in children with ADHD (26). Studies showed that moderate- to high-intensity exercise was more likely to improve emotional regulation and self-efficacy in children with ADHD, while other findings suggested that high-intensity exercise may exacerbate anxiety, depression, and mood disorders in this population (27, 28). These inconsistencies may arise from variations in study design, sample sizes, and the limited number of included studies. Further meta-analytic research is needed to elucidate the effects of different types of physical exercise, their frequency, intensity, and their differential impacts on anxiety, depression, and emotional regulation in children with ADHD (29).

Building upon this foundation, this meta-analysis aims to evaluate the efficacy of physical exercise in addressing anxiety, depression, and emotional challenges in children with ADHD. Additionally, it investigates the impact of various intervention parameters, including exercise type, intensity, duration, frequency, and overall period, on outcome measures. This study endeavors to identify optimal intervention dosages and their corresponding effects, with the objective of providing more precise evidence-based guidance for clinical practice.

## 2 Methods

This systematic review was prospectively registered with the National Institute for Health Research website PROSPERO. The protocol details are accessible at https://www.crd.york.ac.uk/prospero/RecordID=CRD42024571577.

### 2.1 Search strategy

The study strictly followed the PRISMA Statement (Preferred Reporting Items for Systematic Reviews and Meta-Analyses) (30), ensuring a rigorous and standardized reporting methodology. The included literature was thoroughly compiled and analyzed in accordance with the standards outlined in the International Systematic Review Writing Guidelines (31).

A comprehensive literature search was conducted across multiple databases, including Embase, PubMed, Web of Science (WOS), The Cochrane Library, Wanfang Data, VIP Information, and China National Knowledge Infrastructure (CNKI). The search encompassed all publications from the inception of each database through July 31, 2024. Two researchers (YS and SJ) independently performed the literature retrieval. In instances of disagreement, a third researcher was consulted to help resolve the issue and reach a consensus. To ensure a thorough and comprehensive search, the included literature and relevant review references were also examined. The detailed search strategy is provided in Supplementary File S1.

### 2.2 Inclusion criteria

The inclusion criteria were as follows: (1) Studies involving patients aged ≤18 years and diagnosed with ADHD by professionals based on recognized criteria (Diagnostic and Statistical Manual of Mental Disorders or other relevant ADHD diagnostic scales) (32, 33) were included. No restrictions were set on the subtype of ADHD, gender, family background, economic status, level of intelligence, country, or ethnicity of origin of ADHD. (2) The experimental group underwent only exercise intervention, which could include either single or combined interventions of exercise. The control group received usual care and health education, with no additional interventions. (3) Only RCTs were included in the analysis. (4) The mean and standard deviation of anxiety, depression, and emotional regulation were obtainable directly or indirectly in the experimental and control groups before and after the intervention. (5) The baseline data exhibited no significant differences between the experimental and control groups.

### 2.3 Exclusion criteria

The exclusion criteria were as follows: (1) Studies involving patients with autism, Tourette syndrome, epileptic disorders, or other comorbid conditions, (2) studies in which the intervention that did not involve a singular or combined exercise program, (3) studies with incomplete or unextractable data, (4) reviews, commentaries, case studies, cohort studies, book chapters, or duplicate publications, (5) studies with poor methodological quality [Physiotherapy Evidence Database (PEDro) scores < 4] (34), and (6) non-randomized controlled trials.

### 2.4 Definitions and assessment tools for primary outcome indicators

Anxiety is an emotional disorder primarily characterized by excessive worry, restlessness, fear, and physical symptoms, such as palpitations, sweating, and insomnia (35). Assessment instruments for anxiety include the Behavioral Assessment System for Children-T (BASC-T), Child Behavior Checklist (CBCL), Conners Rating Scales (CRS), Youth Self-Report Scales (YSRS), and Beck Anxiety Inventory (BAI).

Depression is characterized by a persistent feeling of sadness and/or a diminished interest or pleasure in activities. In children with ADHD, depression frequently manifests as irritability rather than low mood. It often co-occurs with anxiety, presenting more physical discomfort, and may include psychiatric symptoms such as hallucinations (36). Several assessment tools were utilized to evaluate depression in children, including the CRS, BASC-T, CBCL, and Children's Depression Inventory (CDI).

Emotional dysregulation symptoms in ADHD are frequently characterized by irritability, frustration, and rapid mood swings. The intensity or frequency of these symptoms often appears disproportionate to the individual's environment, age, and developmental stage (37). Various assessment tools were utilized to evaluate emotional dysregulation, including the CRS, Profile of Mood States (POMS), Short Mood and Feelings Questionnaire (SMFQ), Kinder Lebensqualität Fragebogen (KINDL), and Pediatric Quality of Life Inventory (PedsQL).

### 2.5 Literature screening and data extraction

Two authors (YS and SJ) independently screened the studies and extracted data based on predefined inclusion and exclusion criteria. In cases of disagreement, the authors discussed and reached a consensus. If consensus could not be achieved, a third author (XW), blinded to the screening process, made the final decision on study inclusion. The following data were extracted: basic study characteristics (author, publication year, country), subject information (sample size, age), intervention details (specific measures for both experimental and control groups), intervention dosage (time, frequency, duration), and main outcome indicators. When relevant statistics were not reported, the means and standard deviations were estimated based on the sample size, median, range (minimum and maximum), and interquartile range (38). All studies included in this research were published in peer-reviewed journals. In cases where original data were presented only in visual formats (e.g., pictures) or remained unavailable despite contacting the original authors via email, the data were extracted using a chart extraction tool. If a single study employed two or more assessment methods for outcome indicators, only the results of the most commonly used method were extracted, or subgroup data were combined to minimize the risk of double counting and reduce anomaly weighting. The Cohen's kappa value for both researchers was 0.94, indicating a high level of agreement.

### 2.6 Quality assessment of literature

Two assessors (YS and MQ) evaluated the quality of the included studies utilizing the PEDro scale (39) and assessed the risk of bias in the studies employing the Cochrane risk-of-bias tool (latest version, ROB-2) (40).

The PEDro scale an 11-item assessment tool used to evaluate the methodological design and statistical reliability of clinical studies. The “eligibility criteria” item is excluded from the scoring system. Each item is scored with 1 point if it meets the specified criterion, and0 points if it does not. The total score was used to categorize studies into quality levels: scores below 4 points indicated poor quality, scores between 4 and 5 represented fair quality, scores between 6 and 8 were deemed good quality, and scores between 9 and 10 were classified as excellent quality. The Cochrane ROB-2 tool complemented the PEDro scale by assessing biases in the design and outcomes of RCTs. This tool categorizes studies as having “low risk,” “some concern,” or “high risk” of bias.

### 2.7 Quality assessment of evidence

The GRADE profiler system (41) was employed to assess the quality of evidence for the outcome indicators. This assessment incorporates five downgrading factors: publication bias, inconsistency, imprecision, indirectness, and risk of bias. The evidence was classified into four levels: high (no downgrading), moderate (downgraded by one level), low (downgraded by two levels), and very low (downgraded by three levels). Two researchers independently conducted the quality assessment. In cases of discrepancy, a third researcher was consulted to reach a consensus through discussion.

### 2.8 Statistical analyses

Stata 18 software was employed for effect size combination, subgroup analysis, sensitivity analysis, and publication bias testing. The standardized mean difference (SMD) and 95% confidence interval (CI) were utilized to combine effect sizes, accounting for variations in testing methods and measurement units. SMD values were categorized as fair (<0.2), small (0.2–<0.5), moderate (0.5–<0.8), and large (≥0.8). Heterogeneity was assessed using Higgins' *I*^2^ statistic, with *I*^2^ ≤ 25% indicating no heterogeneity, 25% < *I*^2^ ≤ 50% low heterogeneity, 50% < *I*^2^ ≤ 75% moderate heterogeneity, and *I*^2^ > 75% high heterogeneity. Studies were considered homogeneous and analyzed using a fixed-effect model if I2 < 50% and *p* > 0.1. Further determination of heterogeneity sources was necessary if I2 ≥ 50% and *p* < 0.1. Sensitivity analyses were conducted when *I*^2^ exceeded 50% to assess the robustness of merged results. Publication bias was evaluated using Stata 18.0 software, employing funnel plots and Egger's test. The “trim and fill” method was used to further evaluate the stability of meta-analysis results if publication bias was indicated. Publication bias was not assessed for fewer than five studies due to low test efficacy. The significance level for the meta-analysis was set at *p* ≤ 0.05.

## 3 Results

### 3.1 Search results

**Figure 1:**
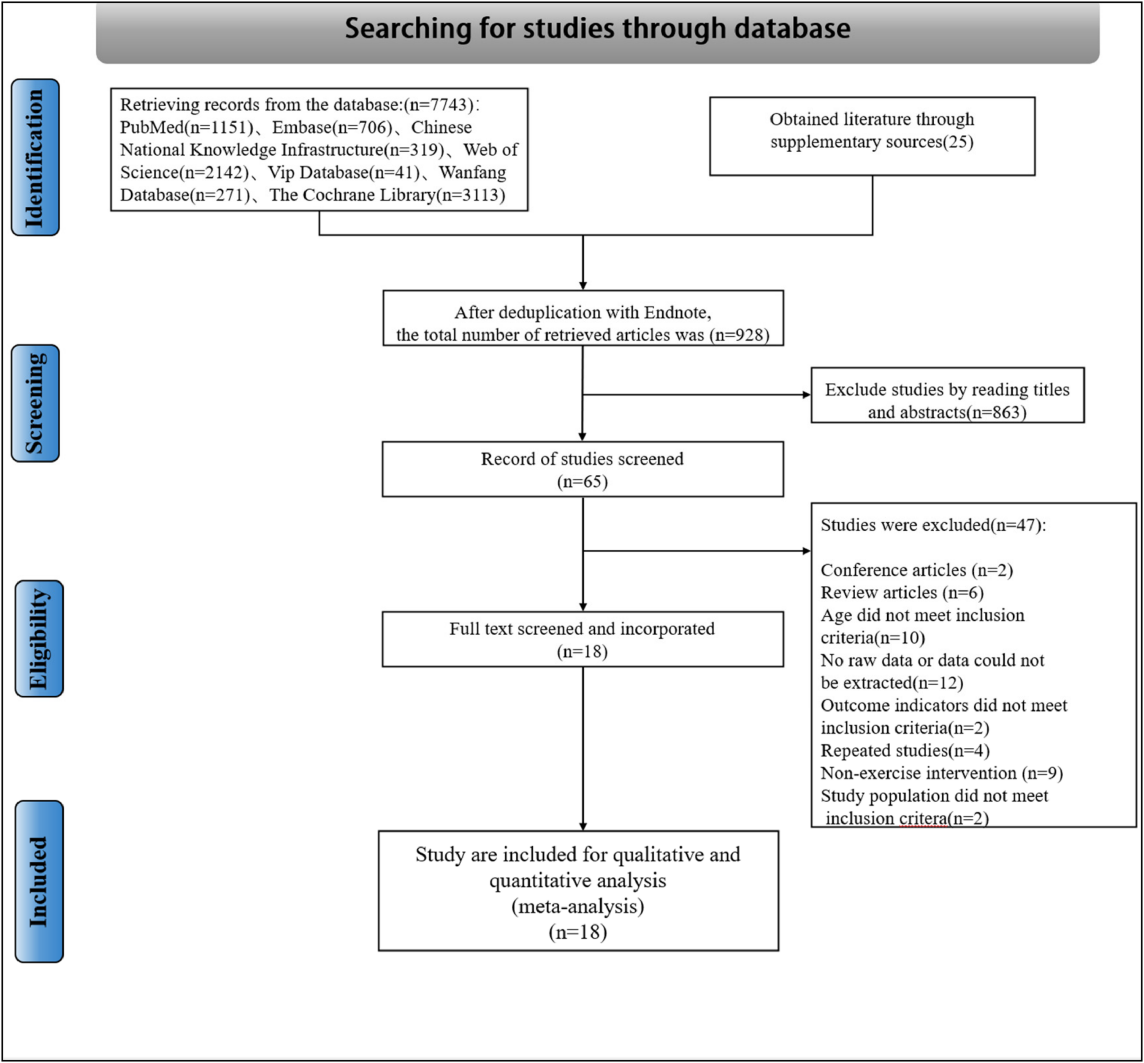
Flow chart of literature selection and inclusion.

A comprehensive literature search yielded 7,743 articles from various databases: Embase (*n* = 706), PubMed (*n* = 1,151), WOS (*n* = 2,142), The Cochrane Library (*n* = 3,113), Wanfang Data (*n* = 271), VIP Information (*n* = 41), and CNKI (*n* = 319). An additional 25 articles were manually identified. Following the initial screening of titles and abstracts, 65 papers were selected for further evaluation. The subsequent full-text review resulted in the exclusion of 47 articles due to various reasons: Twelve lacked relevant endpoint data, two did not meet endpoint criteria, nine employed non-conforming intervention methods, four were duplicates, two were conference publications, six were review articles, ten did not meet age-related inclusion criteria, and two did not meet population-related inclusion criteria. Ultimately, eighteen articles were included in the final analysis. Figure 1 provides a detailed illustration of the literature screening process.

### 3.2 Basic information and intervention characteristics of the included studies

The analysis included 18 RCTs (27–29, 42–56), encompassing 830 participants. The subjects were predominantly children diagnosed with ADHD, ranging from 5 to 14 years of age, with studies published between 2004 and 2023. All included studies implemented an exercise intervention for the experimental group, while the control group received conventional treatments or reading interventions. The physical exercise interventions were primarily classified into two categories: monotypic exercise interventions (55.6%) and mixed exercise interventions (44.4%). Monotypic exercise modes involve a single form of physical activity, whereas mixed exercise modes incorporate multiple forms. The control group underwent conventional rehabilitation, medication, social-behavioral interventions, and a structured educational approach combined with auditory integration training. Intervention frequency varied from 1 to 7 sessions per week, spanning 1 to 20 weeks, with individual sessions lasting between 10 and 90 min. Exercise intensities were categorized as low intensity (44.4%), moderate intensity (33.3%), and high intensity (22.3%). No adverse events were reported in any of the included studies. Additional details are provided in Table 1.

**Table 1:** Intervention characteristics of included studies.

Literature included	Countries	Sample (E/C)	Diagnostic standards	Mean age/years (E/C)		Intervention type		Intervention dose				Outcome indicator
				Experimental group	Control group	Experimental group (The type of exercise)	Control group	Period (week)	Frequency (times/week)	Duration (minute/times)	Exercise intensity	
García-Gómez et al. (44)	Spain	9/5	DSM-IV-TR	10.65 ± 1.50	10.20 ± 2.38	Equestrian therapy (Monotypic)	Sit quietly (unclear amount medicated)	12	2	45	MI	BASC-T^a^
Jensen et al. (51)	Australia	11/8	DSM-IV	10.63 ± 1.78	9.35 ± 1.70	Yoga (Monotypic)	Standard treatment (unclear amount medicated)	20	1	60	LI	CRS^a^
Lufi et al. (43)	Israel	15/17	DSM-IV-TR	11.05 ± 1.54	10.65 ± 1.42	Group-AE(Mixed)	Routine treatment	20	1	90	LI	YSRS^a^
Oh et al. (47)	Korea	17/17	DSM-IV-TR	8.30 ± 1.48	8 ± 1.70	Hippotherapy (Monotypic)	Pharmaco therapy	12	2	60	LI	CBCL^a^
Pan et al. (46)	China	16/16	DSM-IV	8.93 ± 1.49	8.87 ± 1.56	Table tennis (Monotypic)	Standard treatment (56% medicated)	12	2	70	LI	CBCL^a^
Silva et al. (48)	Brazil	10/10	DSM-IV	12 ± 2	12 ± 1	Swimming (Monotypic)	No treatment	8	2	45	MI	BAI^b^, CDI^b^
Yucui Li et al. (56)	China	45/45	MPD	9.32 ± 1.11	9.21 ± 1.13	Aerobic exercise (Mixed)	Routine treatment	16	2	50	LI	CRS^a^
Sabzi et al. (49)	Iran	20/20	MPD	9.6 ± 0.5	9.5 ± 0.3	Water treadmill (Monotypic)	Routine treatment	8	3	30	MI	CRS^a^
Hattabi et al. (50)	Tunis	20/20	DSM-IV	9.95 ± 1.31	9.75 ± 1.33	Swimming (Monotypic)	No treatment	12	3	90	MI	CBCL^a^
Xiaolin Li et al. (57)	China	12/11	DSM-IV	8.92 ± 1.51	8.28 ± 1.42	Qizi Attention Gong (Mixed)	Cognitive and attention training	8	1	60	LI	CRS^a^
Fagui Lin et al. (54)	China	20/20	ADHD-CN	5.0 ± 2.5	5.2 ± 2.3	Sling exercise training (Monotypic)	Routine treatment	12	5	45	LI	CRS^a^
Yumin Chen et al. (55)	China	18/18	DSM-IV	8.48 ± 0.52	8.51 ± 0.50	Tai ji (Monotypic)	Routine treatment	16	3	60	LI	CBCL^a^
Ahmed et al. (42)	Egypt	42/42	CRS-MV	13.9 ± 1.6	13.8 ± 1.7	Aerobic exercise (Mixed)	No treatment	10	3	40–50	MI	CRS^a^
Bigelow et al. (28)	Canada	16/16	MPD	11.38 ± 1.5	11.38 ± 1.5	Cycle ergometer (Monotypic)	Reading education	1	1	10	MI	POMS^b^
Hoza et al. (27)	American	49/45	DSM-IV-TR	6.83 ± 0.96	6.83 ± 0.96	Aerobic exercise (Mixed)	Sedentary activity	12	5	31	HI	CRS^a^
Meßler et al. (45)	Germany	14/14	ICD-10	11 ± 1	11 ± 1	HIIT (Mixed)	Low- to moderate-intensity exercise program	3	3	30	HI	KINDL^a^
Wymbs et al. (29)	American	78/52	DSM-5	9.65 ± 2.46	8.64 ± 1.84	High-intensity group exercise (Mixed)	Low-intensity exercise	2	7	35–40	LHI	SMFQ^b^
Zhang et al. (52)	China	21/21	DSM-5	8.51 ± 1.54	9.09 ± 1.27	Fitness (Mixed)	regular physical education classes	12	3	60	HI	PedsQL^a^

BASC-T, Behavioral Assessment System for Children-T; CRS, Conners Rating Scales; YSRS, Youth Self-Report Scales; CBCL, Child Behavior Checklist; BAI, Beck Anxiety Inventory; POMS, Profile of Mood States; KINDL, Kinder Lebensqualität Fragebogen; SMFQ, Short Mood and Feelings Questionnaire; PedsQL, Pediatric Quality of Life Inventory; LI, low intensity; MI, moderate intensity; HI, high intensity; LHI, low high intensity. ADHD-CN-SE, Guidelines for the Prevention and Treatment of Attention Deficit Hyperactivity Disorder in China, Second Edition; CRS-MV, Conners Rating Scale-modified version; MPD, medical professionals diagnose; MLPA, moderate low intensity physical activity; AE, aerobic exercise CDI, Children's Depression Inventory; R, randomized; C, control group.

^a^ The subcomponent score of the measures.

^b^ The total score of the measures.

### 3.3 Quality evaluation of included studies

All 18 RCTs included in this review met the following criteria: “participant selection criteria specified,” “random assignment of participants to groups,” “baseline similarity,” “participation rate >85%,” “intention-to-treat analysis,” “analysis of statistical results between groups,” and “the study reported point and variability measures for at least one main outcome,” as detailed in Table 2. Among these, seven studies (27, 45, 47–50, 52) achieved “allocation concealment,” one study implemented blinding for outcome assessment, and none reported a “participation rate ≤15%.” Regarding the PEDro scale, the 18 studies scored between 6 and 8, with a mean score of 6.4. No studies of low methodological quality were identified, suggesting that the overall methodological quality was satisfactory.

**Table 2:** Methodological quality of the included studies.

Included study	A1	A2	A3	A4	A5	A6	A7	A8	A9	A10	A11	Toal score
García-Gómez et al. (44)	Y	Y	N	Y	N	N	N	Y	Y	Y	Y	6/10
Jensen et al. (51)	Y	Y	N	Y	N	N	N	Y	Y	Y	Y	6/10
Lufi et al. (43)	Y	Y	N	Y	N	N	N	Y	Y	Y	Y	6/10
Oh et al. (47)	Y	Y	Y	Y	N	N	N	Y	Y	Y	Y	7/10
Pan et al. (46)	Y	Y	N	Y	N	N	N	Y	Y	Y	Y	6/10
Silva et al. (48)	Y	Y	Y	Y	N	N	N	Y	Y	Y	Y	7/10
Yucui Li et al. (56)	Y	Y	N	Y	N	N	N	Y	Y	Y	Y	6/10
Sabzi et al. (49)	Y	Y	Y	Y	N	N	N	Y	Y	Y	Y	7/10
Hattabi et al. (50)	Y	Y	Y	Y	N	N	N	Y	Y	Y	Y	7/10
Xiaolin Li et al. (57)	Y	Y	N	Y	N	N	N	Y	Y	Y	Y	6/10
Fagui Lin et al. (54)	Y	Y	N	Y	N	N	N	Y	Y	Y	Y	6/10
Yumin Chen et al. (55)	Y	Y	N	Y	N	N	N	Y	Y	Y	Y	6/10
Ahmed et al. (42)	Y	Y	N	Y	N	N	N	Y	Y	Y	Y	6/10
Bigelow et al. (28)	Y	Y	N	Y	N	N	N	Y	Y	Y	Y	6/10
Hoza et al. (27)	Y	Y	Y	Y	N	N	Y	Y	Y	Y	Y	8/10
Meßler et al. (45)	Y	Y	Y	Y	N	N	N	Y	Y	Y	Y	7/10
Wymbs et al. (29)	Y	Y	N	Y	N	N	N	Y	Y	Y	Y	6/10
Zhang et al. (52)	Y	Y	Y	Y	N	N	N	Y	Y	Y	Y	7/10

Abbreviations: N, no; Y, yes; A1, participant selection criteria specified; A2, random assignment of participants to groups; A3, hidden assignment; A4, groups were similar at baseline; A5, all participants were blind; A6, all therapists were blind; A7, all assessors were blind; A8, measurement of at least one of the main outcomes was obtained from more than 85% of the participants; A9, intention-to-treat analysis was conducted; A10, results of statistical comparisons between groups for at least one main outcome were reported; A11, the study reported point and variability measures for at least one main outcome.

**Figure 2:**
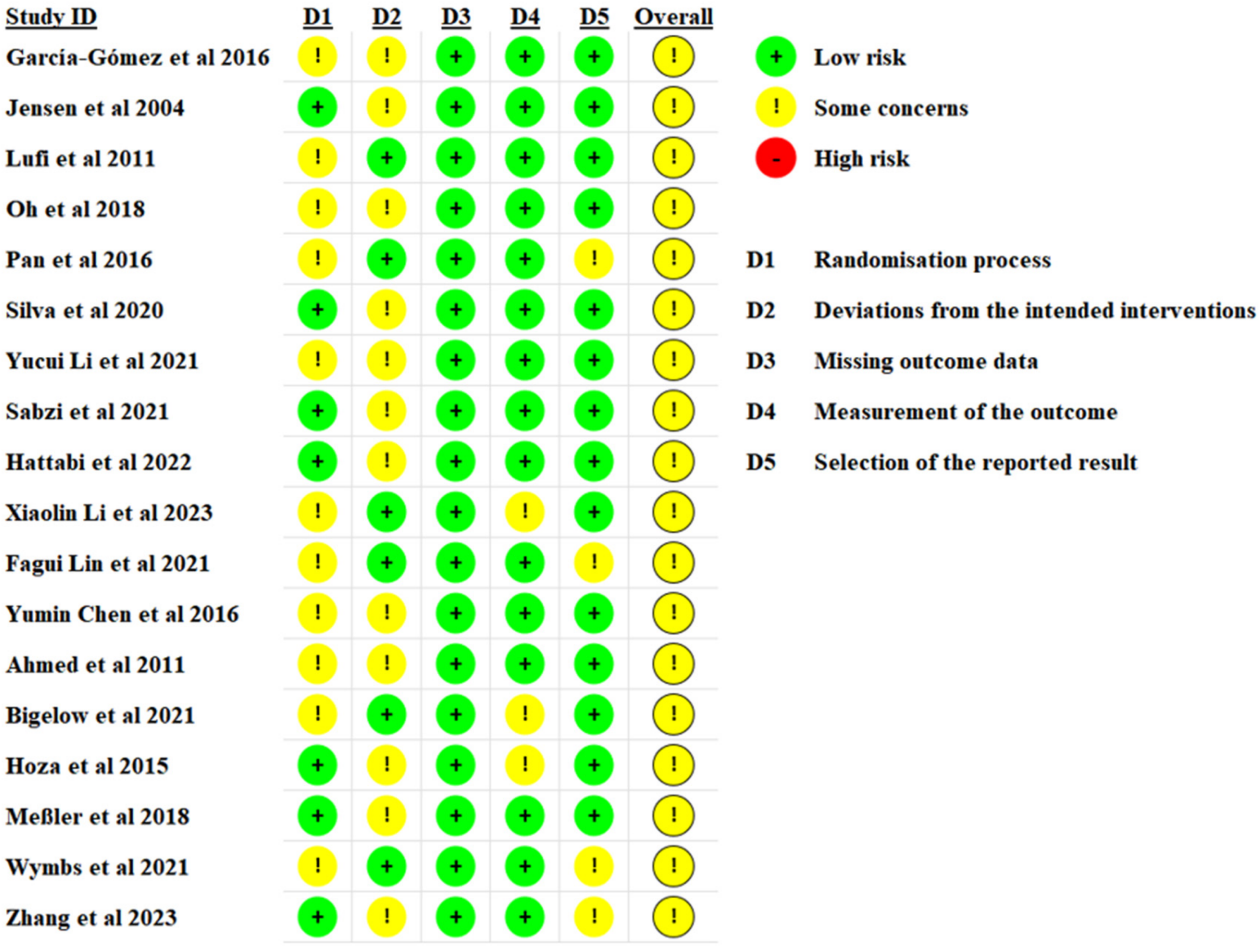
Assessment of methodological quality included in the study.

Based on the ROB-2 assessment tool, seven studies clearly delineated the randomization process and were categorized as low risk for bias. Six studies adequately described the intended intervention and were consequently deemed at low risk of bias. Eighteen studies demonstrated no bias due to missing outcome data, fifteen studies showed no bias in outcome measurement, and fourteen studies exhibited no bias in outcome reporting selection. The final quality ratings for the included literature were as follows: B for eighteen documents, with no documents receiving A or C ratings (Figure 2). Two independent reviewers (SY and SJ) independently assessed the quality of the literature during the evaluation process.

### 3.4 Meta-analysis

#### 3.4.1 Summary

**Figure 3:**
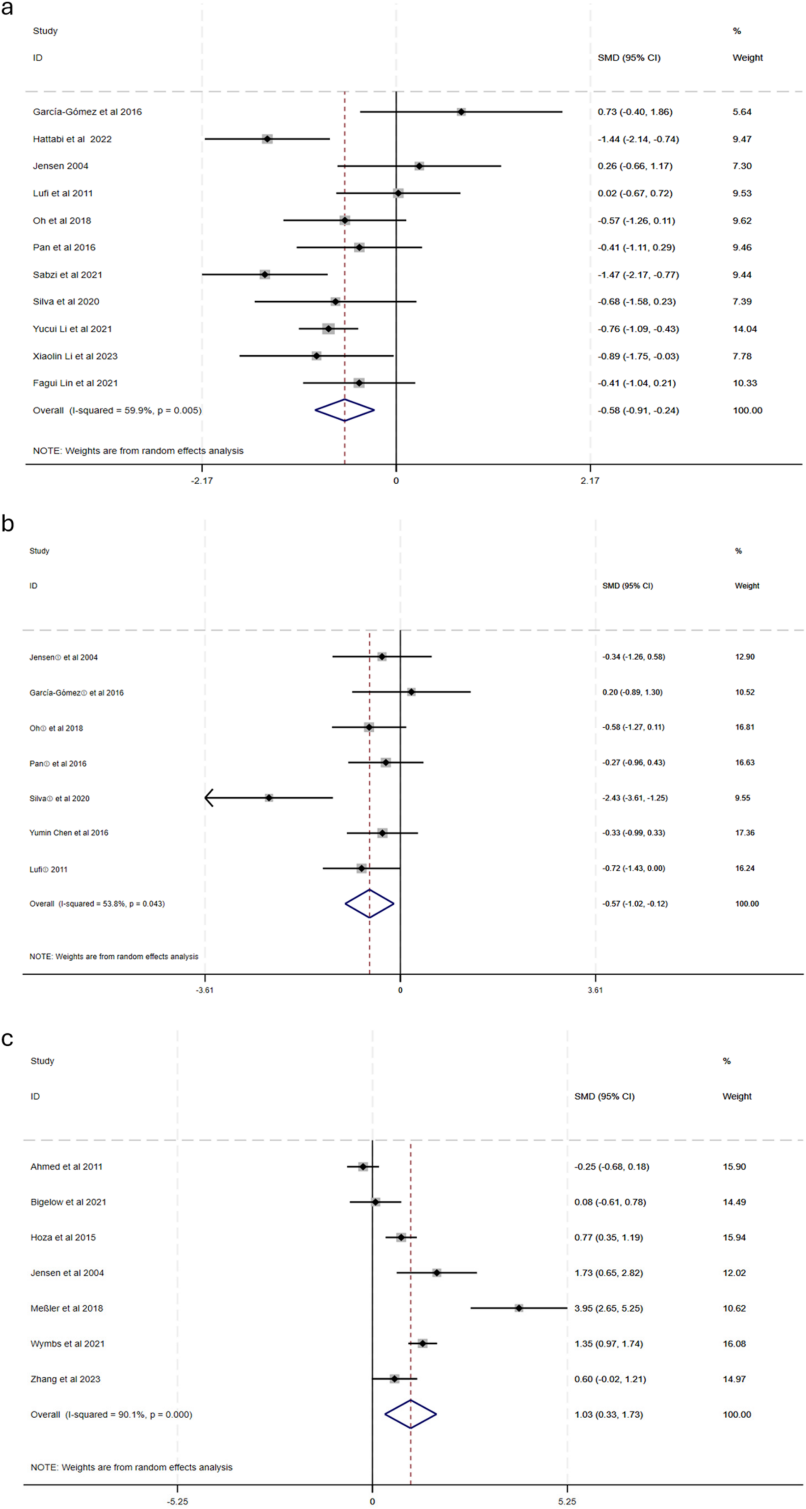
Forest diagram of the effect of physical activity on anxiety, depression and emotion in children with ADHD [**(a)** anxiety, **(b)** depression; **(c)** emotion].

This research evaluated 18 studies (*n* = 830) investigating the impact of physical exercise on anxiety, depression, and emotional states in patients with ADHD. The meta-analysis results for anxiety, depression, and emotional regulation in the physical exercise group are presented in Figure 3. Eleven studies (43, 44, 48–51, 53, 54, 56) reported the effects of physical exercise on anxiety in ADHD patients.

This systematic review analyzed 18 studies (*n* = 830) investigating the impact of physical exercise on anxiety, depression, and emotional regulation in individuals with ADHD. The meta-analysis demonstrated significant improvements across all three outcomes:

**Anxiety**: Eleven studies (46, 52, 55, 56, 58–64) evaluated the effects of physical exercise on anxiety in individuals with ADHD. The analysis revealed moderate heterogeneity (X^2^ = 24.96, *I*^2^ = 59.9%, *p* = 0.005), necessitating the application of a random-effects model. The pooled effect size was statistically significant [SMD = −0.58, 95% CI: (−0.91, −0.24), *p* < 0.05], indicating a moderate reduction in anxiety levels.

**Depression**: Seven studies (55, 56, 60, 62–65) examined the impact of exercise on depressive symptoms. Moderate heterogeneity was observed (X^2^ = 12.9, *I*^2^ = 53.8%, *p* = 0.043), necessitating the use of a random-effects model. The meta-analysis demonstrated a significant reduction in depression [SMD = −0.57, 95% CI: (−1.02, −0.12), *p* < 0.05].

**Emotional regulation:** Seven studies (26, 35–37, 54, 56, 66) investigated the impact of physical activity on emotional regulation. Due to high heterogeneity (X^2^ = 60.40, *I*^2^ = 90.1%, *p* < 0.001), a random-effects model was employed. The analysis revealed a large positive effect [SMD = 1.03, 95% CI: (0.33, 1.73), *p* < 0.05], suggesting substantial improvements in emotional regulation.

#### 3.4.2 Subgroup analyses

##### 3.4.2.1 Subgroup analyses of anxiety

Subgroup analyses (Table 3) were performed to investigate the influence of five moderating variables on the efficacy of physical exercise interventions: exercise type, intervention period, frequency, duration, and intensity. The findings are summarized as follows:

**Table 3:** Subgroup analysis of the effects of physical exercise on anxiety.

Moderating variable		Number of studies	Sample size	Heterogeneity			Effect		
				*Q*	*p*	*I*^2^%	SMD	95% CI	*p*
The type of exercise	Monotypic	8	239	21.22	0.003	68.69	−0.55	(−1.019, −0.077)	0.023
	Mixed	3	205	4.40	0.111	54.72	−0.55	(−1.058, −0.048)	0.032
Period/week	≤12	8	243	17.18	0.016	59.54	−0.68	(−1.095, −0.272)	0.001
	>12	3	201	7.35	0.025	70.15	−0.25	(−0.896, 0.389)	0.439
Frequency (time/week)	1	3	74	3.85	0.146	47.81	−0.19	(−0.819, 0.440)	0.555
	2	5	250	6.84	0.144	30.36	−0.51	(−0.852, −0.164)	0.004
	≥3	3	120	6.55	0.038	68.70	−1.07	(−1.752, −0.391)	0.002
Intervention duration/min	≤45	4	114	11.83	0.008	76.84	−0.51	(−1.323, 0.300)	0.217
	45–60	4	226	4.73	0.193	26.56	−0.59	(−0.944, −0.237)	0.001
	>60	3	104	9.03	0.011	77.85	−0.60	(−1.431, 0.238)	0.161
Exercise intensity	Low intensity	8	344	13.03	0.071	42.63	−0.36	(−0.673, −0.055)	0.021
	Moderate intensity	3	100	2.37	0.306	0.00	−1.24	(−1.663, −0.820)	<0.001

**Type of Physical Exercise:** Monotypic exercise intervention exhibited a significant effect [SMD = −0.55, 95% CI: (−1.019, −0.077), *p* = 0.023]. Similarly, mixed exercise intervention demonstrated a significant effect [SMD = −0.55, 95% CI: (−1.068, −0.048), *p* = 0.032].

**Physical Exercise Intervention Period:** The analysis revealed that short-term interventions demonstrated a significant effect compared to the control group [SMD = −0.68, 95% CI: (−1.096, −0.272), *p* = 0.001]. In contrast, long-term interventions did not exhibit a statistically significant difference compared to the control group [SMD = −0.25, 95% CI: (−0.896, 0.439), *p* = 0.439].

**Physical Exercise Intervention Frequency:** Low-frequency exercise [SMD = −0.19, 95% CI: (−0.819, 0.440), *p* = 0.555] did not demonstrate a significant difference compared to the control group. Conversely, moderate-frequency exercise [SMD = −0.51, 95% CI: (−0.852, −0.164), *p* = 0.004] and high-frequency exercise [SMD = −1.07, 95% CI: (−1.752, −0.391), *p* = 0.002] both exhibited significant effects in comparison to the control group.

**Physical Exercise Intervention Duration:** Neither short-duration interventions [SMD = −0.51, 95% CI: (−1.323, 0.300), *p* = 0.217] nor long-duration interventions [SMD = −0.60, 95% CI: (−1.431, 0.238), *p* = 0.161] demonstrated significant differences from the control group. However, medium-duration interventions [SMD = −0.59, 95% CI: (−0.944, −0.237), *p* = 0.001] exhibited a significant effect in comparison to the control group.

**Physical Exercise Intervention Intensity:** Low-intensity exercise [SMD = −0.36, 95% CI: (−0.673, −0.055), *p* = 0.021] and moderate-intensity exercise [SMD = −1.24, 95% CI: (−1.663, −0.820), *p* < 0.001] both demonstrated significant improvements in outcomes when compared to the control group.

##### 3.4.2.2 Subgroup analyses of depression

Subgroup analyses (Table 4) were conducted based on five moderating variables: type of exercise, intervention period, frequency, duration, and intensity. The following summarizes the results:

**Table 4:** Subgroup analysis of the effects of physical exercise on depression.

Moderating variable		Number of studies	Sample size	Heterogeneity			Effect		
				*Q*	*p*	*I*^2^%	SMD	95% CI	*p*
The type of exercise	Monotypic	6	155	15.49	0.008	74.38	−0.55	(−1.120, 0.026)	0.061
	Mixed	1	32	0.00	<0.001	0.00	−0.70	(−1.396, −0.007)	0.041
Period/week	≤12	4	100	12.61	0.006	82.11	−0.70	(−1.694, 0.291)	0.166
	>12	3	87	2.74	0.254	19.59	−0.46	(−0.874, −0.041)	0.031
Frequency (time/week)	1	2	51	2.74	0.098	63.51	−0.55	(−1.099, −0.007)	0.047
	2	4	100	12.61	0.006	82.11	−0.70	(−1.694, 0.291)	0.166
	≥3	1	36	−0.00	<0.001	0.00	−0.32	(−0.968, 0.318)	0.322
Intervention duration/min	≤45	2	34	10.72	0.001	90.67	−1.06	(−3.530, 1.411)	0.400
	45∼60	3	89	2.10	0.349	0.00	−0.41	(−0.824, −0.004)	0.048
	>60	2	64	0.77	0.379	0.00	−0.47	(−0.960, 0.013)	0.056
Exercise intensity	Low intensity	6	167	4.24	0.515	0.00	−0.39	(−0.685, −0.085)	0.012
	Moderate intensity	1	20	0.00	<0.001	0.00	−2.33	(−3.436, −1.222)	<0.001

**Type of Physical Exercise Intervention: Monotypic exercise intervention** demonstrated a significant difference in comparison to the control group [SMD = −0.55, 95% CI: (−1.120, −0.026), *p* = 0.061]. **Mixed exercise intervention** did not exhibit a significant difference [SMD = −0.70, 95% CI: (−1.396, −0.007), *p* = 0.041].

**Physical Exercise Intervention Period: Short-term interventions** [SMD = −0.70, 95% CI: (−1.694, 0.291), *p* = 0.166] did not demonstrate a significant difference compared to the control group. **Long-term interventions** [SMD = −0.46, 95% CI: (−0.874, −0.041), *p* = 0.031] exhibited a statistically significant difference in comparison to the control group.

**Physical Exercise Intervention Frequency: Low-frequency exercise** [SMD = −0.55, 95% CI: (−1.099, −0.007), *p* = 0.047] demonstrated a statistically significant difference compared to the control group. **Moderate-frequency exercise** [SMD = −0.70, 95% CI: (−1.694, 0.291), *p* = 0.166] and **high-frequency exercise** [SMD = −0.32, 95% CI: (−0.968, 0.318), *p* = 0.322] did not exhibit significant differences compared to the control group.

**Physical Exercise Intervention Duration: Short-duration interventions** [SMD = −1.06, 95% CI: (−3.530, 1.411), *p* = 0.400] and **long-duration interventions** [SMD = −0.47, 95% CI: (−0.960, 0.013), *p* = 0.056] did not demonstrate significant differences from the control group. **Medium-duration interventions** [SMD = −0.41, 95% CI: (−0.824, −0.004), *p* = 0.048] exhibited a significant difference compared to the control group.

**Physical Exercise Intervention Intensity: Low-intensity exercise** [SMD = −0.39, 95% CI: (−0.685, −0.085), *p* = 0.012] and **moderate-intensity exercise** [SMD = −2.33, 95% CI: (−3.436, −1.22), *p* < 0.001] both demonstrated statistically significant differences in comparison to the control group.

##### 3.4.2.3 Subgroup analyses of emotion regulation

Subgroup analyses (Table 5) were performed to investigate the impact of various moderating variables on the efficacy of physical exercise interventions. The findings are summarized as follows:

**Table 5:** Subgroup analysis of the effects of physical exercise on emotion regulation.

Moderating variable		Number of studies	Sample size	Heterogeneity			Effect		
				*Q*	*p*	*I*^2^%	SMD	95% CI	*p*
The type of exercise	Monotypic	2	51	6.40	0.011	84.39	0.82	(−0.721, 2.363)	0.297
	Mixed	5	379	54.73	<0.001	96.76	1.18	(0.078, 2.437)	0.006
Period/week	≤12	6	411	58.23	<0.001	95.94	0.99	(−0.079, 2.055)	0.070
	>12	1	19	0.00	<0.001	0.00	1.66	(0.640, 2.674)	0.001
Frequency (time/week)	1	2	51	6.40	0.011	84.39	0.82	(−0.721, 2.363)	0.297
	>2	5	379	54.73	<0.001	96.76	1.18	(0.078, 2.437)	0.006
Intervention duration/min	≤45	4	287	31.47	<0.001	96.55	1.44	(0.066, 2.941)	0.001
	45–60	3	143	13.78	0.001	87.53	0.58	(−0.461, 1.627)	0.273
Exercise intensity	Low intensity	1	19	0.00	<0.001	0.00	1.66	(0.640, 2.674)	0.001
	Moderate intensity	3	144	37.64	<0.001	97.40	1.17	(−1.349, 3.682)	0.363
	High intensity	3	267	6.14	0.047	66.65	0.93	(0.476, 1.392)	<0.001

**Type of Physical Exercise Intervention: Monotypic exercise intervention** [SMD = 0.82, 95% CI: (−0.721, 2.363), *p* = 0.297] did not demonstrate a statistically significant difference compared to the control group. In contrast, **mixed exercise intervention** [SMD = 1.18, 95% CI: (0.078, 2.437), *p* = 0.006] exhibited a statistically significant difference compared to the control group.

**Physical Exercise Intervention Period: Short-term interventions** [SMD = 0.99, 95% CI: (−0.079, 2.055), *p* = 0.070] did not demonstrate a statistically significant difference in comparison to the control group. **Long-term interventions** [SMD = 1.66, 95% CI: (0.640, 2.674), *p* = 0.001] exhibited a significant difference when compared to the control group.

**Physical Exercise Intervention Frequency: Low-frequency exercise** [SMD = 0.82, 95% CI: (−0.721, 2.363), *p* = 0.297] did not demonstrate a statistically significant difference compared to the control group. Conversely, **moderate-frequency exercise** [SMD = 1.18, 95% CI: (0.078, 2.437), *p* = 0.006] and **high-frequency exercise** [SMD = 1.18, 95% CI: (0.078, 2.437), *p* = 0.006] both exhibited significant effects compared to the control group.

**Physical Exercise Intervention Duration: Short-duration interventions** [SMD = 1.44, 95% CI: (0.066, 2.941), *p* = 0.001] demonstrated a significant difference compared to the control group. **Medium-duration interventions** [SMD = 0.58, 95% CI: (−0.461, 1.627), *p* = 0.273] did not exhibit a statistically significant difference compared to the control group.

**Physical Exercise Intervention Intensity: Low-intensity exercise** [SMD = 1.66, 95% CI: (0.640, 2.674), *p* = 0.001] and **high-intensity exercise** [SMD = 0.93, 95% CI: (0.476, 1.392), *p* < 0.001] demonstrated significant differences compared to the control group. **Moderate-intensity exercise** [SMD = 1.17, 95% CI: (−1.349, 3.682), *p* = 0.363] did not exhibit a significant difference compared to the control group.

### 3.5 Sensitivity analyses

**Figure 4:**
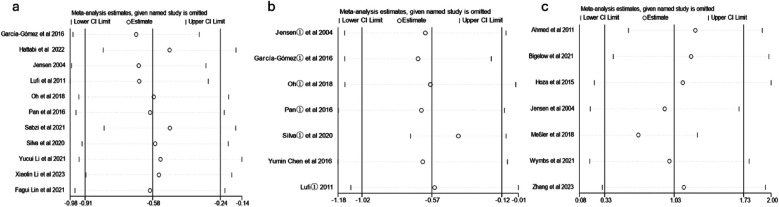
Sensitivity analysis [**(a)** anxiety; **(b)** depression; **(c)** emotion].

To investigate the source of heterogeneity, a sensitivity analysis was performed using Stata 18.0. As illustrated in Figure 4, the combined effect was analyzed by sequentially excluding individual studies. After removing individual studies, the results for anxiety revealed SMD (−0.66 to −0.49), *I*^2^ (53.9%–63.9%), and *p* < 0.05; for depression, SMD (−0.40 to −0.66), *I*^2^ (0.0%–61.4%), and *p* < 0.05; and for emotion regulation, SMD (0.67 to 1.24), *I*^2^ (85.3%–91.7%), and *p* < 0.05. The analysis demonstrated no significant changes before and after the removal of individual studies. The low sensitivity of the study data indicates that the results exhibit a considerable level of reliability and stability. These findings suggest that the combined analysis did not result in substantial alterations, thus demonstrating a degree of robustness.

### 3.6 Publication bias

**Figure 5:**
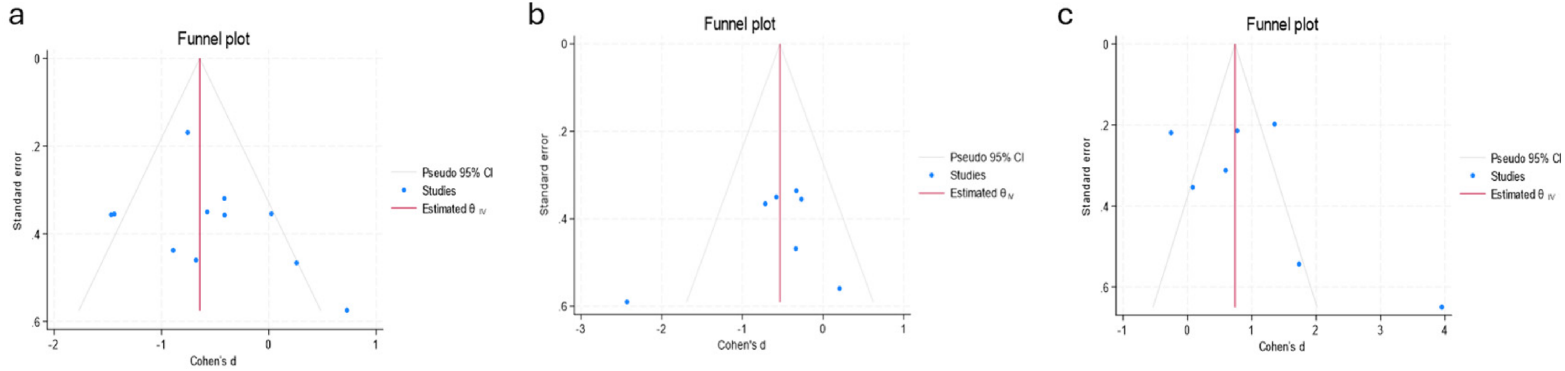
Egger's test results [**(a)** anxiety; **(b)** depression; **(c)** emotion].

Egger's test revealed no significant differences in anxiety (Z = 1.39, *p* > |t| = 0.197 > 0.05), depression (*Z* = −1.11, *p* > |*t*| = 0.317 > 0.05), or emotion regulation (*Z* = 2.54, *p* > |*t*| = 0.052 > 0.05). These results indicated that the findings were relatively robust (Figure 5).

### 3.7 Quality of evidence assessment

**Figure 6:**
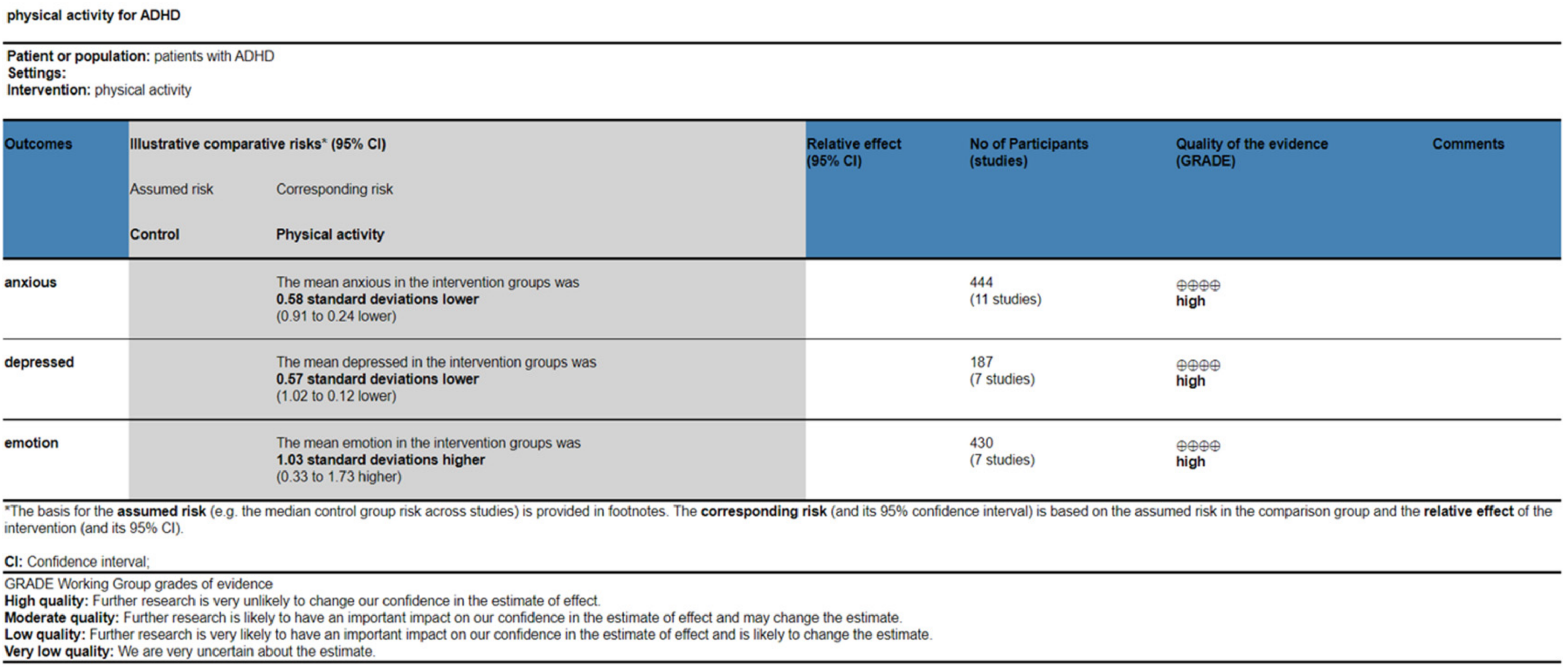
Evidence quality assessment.

The quality of evidence was evaluated utilizing the GRADE profiler methodology. Factors such as publication bias, inconsistency, imprecision, indirectness, and risk of bias were not downgraded. The assessment of the quality of evidence regarding the impact of physical activity on ameliorating anxiety, depression, and emotion regulation in children with ADHD was determined to be of high level, as illustrated in Figure 6.

## 4 Discussion

This study conducted a systematic evaluation and meta-analysis to examine the impact of physical exercise on anxiety, depression, and emotional regulation in children with ADHD. The findings revealed that physical exercise significantly enhanced these aspects in ADHD-affected children. Exercise promotes frontal lobe function development, activates the prefrontal cortex, and stimulates dopamine release, thereby fostering positive emotions and diminishing negative ones (58, 59, 65, 67, 68). Additionally, abnormal catecholamine function has been identified as a crucial pathophysiological mechanism in ADHD. Physical activity influences this mechanism by increasing choline acetate release, which exerts a calming effect and mitigates anxiety, depression, and mood symptoms in children with ADHD (60, 69, 70).

This investigation encompassed 18 RCTs to assess the impact of physical activity on anxiety, depression, and emotional regulation in children with ADHD. The quality of the literature was evaluated using the PEDro scale. The analysis revealed that the 18 RCTs had a mean quality score of 6.4, indicating a high overall quality of literature, with no identified risk of bias. Meta-analysis revealed that physical activity had a moderate effect size (SMD = −0.58) and moderate heterogeneity (*I*^2^ = 59.9%) for anxiety in children with ADHD. Sensitivity analysis demonstrated increased stability of the results. The quality of evidence was deemed high, with no downgrading factors related to imprecision, publication bias, or indirectness observed. However, potential bias in outcome assessment may arise from differences in measurement tools and the lack of consideration for variations in disease duration and medication use among patients in the included studies, warranting careful interpretation.

The type, period, frequency, duration, and intensity of physical activity moderated the improvement of anxiety and depression in children with ADHD. Effectively enhancing the enjoyment of sports participation in children with ADHD is a key factor affecting their ability to increase self-efficacy. A significant difference in anxiety and depression improvement was observed between monotypic and mixed exercise, with mixed exercise showing the greatest effect. This difference may be attributed to mixed exercise being more flexible and engaging than monotypic exercise in program design and implementation, allowing children with ADHD to receive continuous positive emotional feedback during exercise. Exercise load, encompassing duration and intensity, is the most crucial concept in exercise training (61). Frequency, duration, and intensity are specific manifestations of exercise load and are important determinants of exercise intervention effectiveness (62). Regarding the exercise cycle, 8 to 12 weeks of intervention is ideal, with a potential downward trend as the cycle lengthens. For exercise duration, 45–60 min per intervention session is expected to yield greater improvements in anxiety and depression. Insufficient intervention time is unlikely to alter the arousal level of physical functioning or the brain structure and function in children with ADHD. Moreover, the age and pathological characteristics of children with ADHD make prolonged daily exercise interventions nearly impossible to maintain (63). Concerning exercise frequency, more than two sessions per week are expected to produce the greatest intervention effect for anxiety in children with ADHD. For depression, one session per week is anticipated to yield the most significant improvement. Regarding exercise intensity, moderate-intensity interventions produced the greatest benefits for anxiety and depression improvement in children with ADHD, which aligns with the findings of Xie et al. (64).

The current study revealed that physical exercise had a significant effect (SMD = 1.03) with high heterogeneity (*I*^2^ = 90.1%, *p* < 0.001) on improving emotion in children with ADHD. This finding may be attributed to the RCT literature included in this study reporting better homogeneity of effects and more stable results. Subgroup analyses indicated that mixed exercise had a greater ameliorative effect on the mood of children with ADHD compared to monotypic exercise, aligning with the findings of Kiluk et al. (26). Regarding exercise period, interventions for medium- and long-term were most effective; however, higher-quality, large-sample-size RCT trials are necessary to validate whether these benefits persist or increase as the intervention period extends. In terms of exercise frequency, moderate and high frequencies exceeding once per week are expected to produce the greatest intervention effect. Concerning exercise duration, acute physical activity of up to 45 min per session appeared to yield the greatest benefit in improving emotion in children with ADHD, consistent with the findings of Chueh et al. (68), but diverging from those of Fritz et al. (66). This discrepancy may be due to differences in outcome indicator assessment between studies, and the varying specificity and sensitivity of different assessment tools may have influenced the evaluation of mood indicators (71). Regarding exercise intensity, low-intensity physical activity demonstrated the best benefit in improving emotion in children with ADHD. Conversely, no statistically significant difference was observed for moderate-intensity physical activity. This result may be attributed to high-intensity exercise being more likely to produce neurobiological mechanisms similar to those of ADHD medications, which in turn effectively promotes prefrontal cortex development, increases the total amount of hippocampus or gray and white matter in the brain, and improves information transfer between different brain regions and cerebral blood flow, leading to the alleviation of emotional symptoms in children with ADHD (72).

Physical exercise demonstrates a significant and positive effect on reducing anxiety symptoms, mitigating depressive tendencies, and improving emotional regulation in individuals diagnosed with ADHD. These findings underscore the potential of exercise as a complementary therapeutic approach for managing ADHD-related symptoms.

## 5 Strengths and limitations of the study

This study possesses several strengths: (1) The inclusion of only RCTs enhances the study's rigor and the reliability of its results. (2) A comprehensive analysis of moderating variables of motor exercise on anxiety/depression and mood regulation in children with ADHD was conducted, including subgroup analyses of exercise type, duration, intensity, intervention period, and frequency. However, the study also has limitations: (1) Due to insufficient statistical power from small sample sizes, the subgroup analyses were exploratory, and these findings require further confirmation. (2) Variations in exercise intervention parameters among the included studies may have introduced clinical heterogeneity. Future research should identify specific exercise intervention parameters (e.g., type, duration, intervention period, intensity, cycle, and frequency) to determine the optimal exercise intervention program and explore the underlying physiological mechanisms of improvement.

## 6 Conclusions

Physical exercise demonstrated a substantial overall impact on enhancing anxiety, depression, and emotional regulation in children with ADHD, exhibiting a dose-response effect correlated with the period, frequency, duration, and intensity of the exercise. This investigation corroborates the advantageous effects of physical activity in ameliorating anxiety, depression, and emotional regulation in children with ADHD. It presents an additional evidence-based therapeutic approach for the considerable number of children with ADHD who are not appropriate candidates for pharmacological intervention.

## Data Availability

The original contributions presented in the study are included in the article/Supplementary Material, further inquiries can be directed to the corresponding author.
